# Characterization of the CYP3A4 Enzyme Inhibition Potential of Selected Flavonoids

**DOI:** 10.3390/molecules26103018

**Published:** 2021-05-19

**Authors:** Martin Kondža, Mirza Bojić, Ivona Tomić, Željan Maleš, Valentina Rezić, Ivan Ćavar

**Affiliations:** 1 Faculty of Pharmacy, University of Mostar, Matice Hrvatske bb, 88000 Mostar, Bosnia and Herzegovina; martin.kondza@farf.sum.ba (M.K.); ivona.tomic@farf.sum.ba (I.T.); 2 University of Zagreb Faculty of Pharmacy and Biochemistry, Ante Kovačića 1, 10000 Zagreb, Croatia; zeljan.males@pharma.unizg.hr; 3 Farmavita d.o.o., Igmanska 5A, 71000 Sarajevo, Bosnia and Herzegovina; v.rezic@farmavita.ba; 4 Faculty of Medicine, University of Mostar, Zrinskog Frankopana 34, 88000 Mostar, Bosnia and Herzegovina; ivan.cavar@mef.sum.ba

**Keywords:** acacetin, apigenin, chrysin, pinocembrin, inhibition, CYP3A4, flavonoid-drug interaction

## Abstract

Acacetin, apigenin, chrysin, and pinocembrin are flavonoid aglycones found in foods such as parsley, honey, celery, and chamomile tea. Flavonoids can act as substrates and inhibitors of the CYP3A4 enzyme, a heme containing enzyme responsible for the metabolism of one third of drugs on the market. The aim of this study was to investigate the inhibitory effect of selected flavonoids on the CYP3A4 enzyme, the kinetics of inhibition, the possible covalent binding of the inhibitor to the enzyme, and whether flavonoids can act as pseudo-irreversible inhibitors. For the determination of inhibition kinetics, nifedipine oxidation was used as a marker reaction. A hemochromopyridine test was used to assess the possible covalent binding to the heme, and incubation with dialysis was used in order to assess the reversibility of the inhibition. All the tested flavonoids inhibited the CYP3A4 enzyme activity. Chrysin was the most potent inhibitor: *IC*_50_ = 2.5 ± 0.6 µM, *K_i_* = 2.4 ± 1.0 µM, *k_inact_* = 0.07 ± 0.01 min^−1^, *k_inact_*/*K_i_* = 0.03 min^−1^ µM^−1^. Chrysin caused the highest reduction of heme (94.5 ± 0.5% residual concentration). None of the tested flavonoids showed pseudo-irreversible inhibition. Although the inactivation of the CYP3A4 enzyme is caused by interaction with heme, inhibitor-heme adducts could not be trapped. These results indicate that flavonoids have the potential to inhibit the CYP3A4 enzyme and interact with other drugs and medications. However, possible food–drug interactions have to be assessed clinically.

## 1. Introduction

Food is directly linked to the overall health and well-being of humans. Flavonoids are secondary plant metabolites that are consumed through vegetables, fruits, teas, wines, propolis, medicinal plants, and so on. These compounds aid organoleptic characteristics of foods (e.g., color and taste of tea and wine) and are of interest due to biological properties affecting human health [1,2]. As non-essential nutrients, they have received much attention in the last decades. All flavonoids have a similar molecular structure—a phenylbenzo-*γ*-pyrone (rings A, B and C)—to which hydroxyl groups are bound, and these hydroxyl groups can be methylated and glycosylated [3]. Based on different authors and estimations, there are from 4000 to 8000 currently known flavonoids that can be classified into different subgroups based on the structure of ring C (such as flavans, flavanones, flavones, and flavonols) [4]. The consumption of flavonoids from foods varies between societies and countries, i.e., France has a high consumption rate (1193 mg/day) versus the United Kingdom (182 mg/day) [5,6].

**Figure 1:**
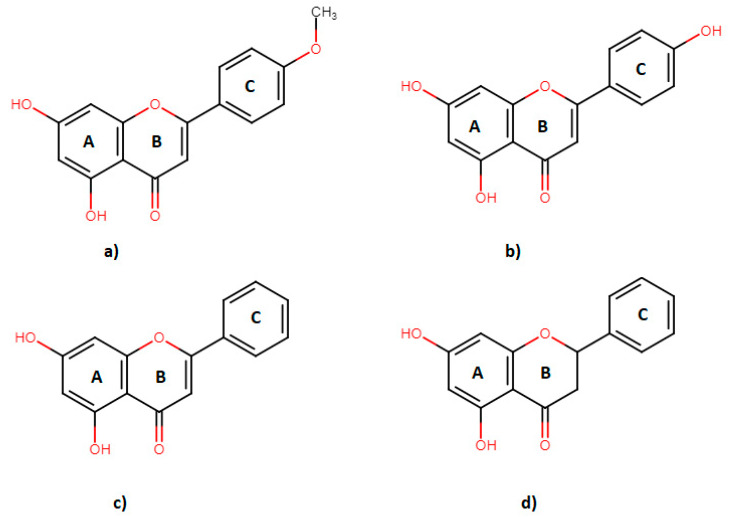
Molecular structures of flavonoids; acacetin (**a**), apigenin (**b**), chrysin (**c**) and pinocembrin (**d**).

Acacetin is an *O*-methylated flavone believed to be connected with the prevention of heart diseases [7], presenting anti-inflammatory [8], anti-plasmodial [9], and anti-proliferative [10,11] effects on tumor cells in vitro. Apigenin has a hydroxyl instead of a methoxy group in its molecular structure (Figure 1). Apigenin is most abundant in parsley—up to of 45.035 µg/g in dried plant [12]. Other food sources of apigenin are chamomile, celery, vine spinach, artichokes, and oregano [13]. Apigenin shows various biological effects, including anti-oxidative [14], anti-hyperglycemic [14], and anti-inflammatory [15] to anti-apoptotic [16]. Chrysin is a 5,7-dihydroxy-flavone (Figure 1), a dietary phytochemical abundantly present in honey and many plant extracts (propolis, blue passion flower) [17]. Chrysin is a potent inhibitor of aromatase (cytochrome P450 19A1 enzyme) [18], showing anti-inflammatory [19] and anti-oxidant [20] effects, as well as the capability to induce apoptosis of cancer cells in vitro [17]. Pinocembrin is a 5,7-dihydroxy-flavanone (Figure 1), which can be mostly found in fruits, vegetables, nuts, seeds, honey, herbs, spices, flowers, tea, and red wine [18,19,20].

Overall, the consumption of flavonoids and foods rich in flavonoids is linked with beneficial effects on human health [21]. However, there is a potential risk that flavonoids can cause interactions with various drugs. A food–drug interaction is a serious safety issue that happens when the pharmacologic effect of a drug is changed by the action of food and/or dietary supplements causing unexpected clinical effects [22]. Drug interactions are responsible for more than 30% of all adverse drug events [23] and about 0.57% of hospital admissions in the United States of America [24]. Flavonoids can cause interactions with certain medications. One of the ways in which flavonoids cause interactions is by inhibiting the enzymes responsible for drug metabolism such as cytochrome P450 enzymes (CYP enzymes), the most significant of which is the CYP3A4 enzyme.

The CYP3A4 enzyme is responsible for the metabolism of 33% of drugs [25]. Besides this, CYP3A4 is involved in the metabolism of many xenobiotics, several of which can act as inhibitors or inducers of its activity. Interactions with other drugs used in therapy and even clinically significant interactions can occur [26]. For example, one of the significant possible interactions is the use of St. John’s wort and oral contraceptives, where the reduced efficacy of oral contraceptives and resulting pregnancies have been noticed [27]. Thus, it is very important to know which compounds can inhibit or induce the activity of the CYP3A4 enzyme. As it has been previously shown, acacetin, apigenin, chrysin, and pinocembrin can cause a statistically significant inhibition of CYP3A4 at 1 µM concentration, using testosterone as a marker substrate of residual enzyme activity [28]. These data suggest that flavonoids have a potential of causing food–drug interactions when foods rich in flavonoids (honey, propolis) are used [29]. As CYP3A4 has a large active site, it is suggested that all inhibition assays are conducted using at least two marker substrates [30].

The aim of this study was to investigate the inhibition kinetic of acacetin, apigenin, chrysin, and pinocembrin using nifedipine as a marker substrate of CYP3A4 enzyme activity. Furthermore, the aim of this study was to investigate the possible covalent binding of flavonoids to the heme part of CYP3A4, as well as to test their possible role in pseudo-irreversible inhibition.

## 2. Results

### 2.1. Enzyme Kinetics

**Figure 2:**
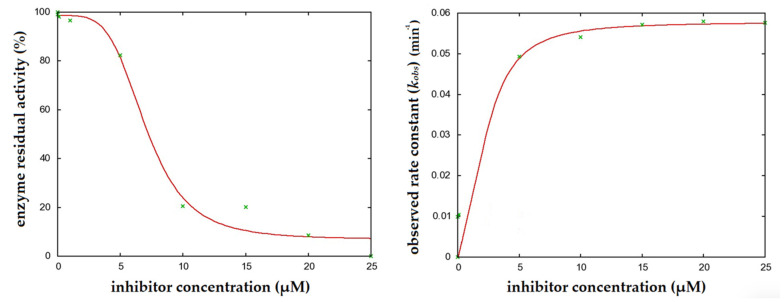
Determination of the enzyme inactivation kinetics parameters of CYP3A4 by chrysin. Experimental data is marked in green and fitted curve in red.

Of all the tested flavonoids, chrysin was the most potent inhibitor of CYP3A4 with a *IC*_50_ value of 2.5 ± 0.6 µM (Figure 2). Pinocembrin, acacetin, and apigenin had *IC*_50_ values of 4.3 ± 1.1, 7.5 ± 2.7, and 8.4 ± 1.1 µM, respectively (Table 1).

*IC*_50_ values are dependent on experimental setup; thus, a complete inhibition kinetic was determined for individual flavonoids using different concentrations of flavonoid incubated for different time periods (vide infra). Chrysin also showed the lowest inhibition constant—*K_i_* value. The inhibition constants of individual flavonoids were tested using nifedipine as a marker substrate, and for acacetin, apigenin, chrysin, and pinocembrin, the following *K_i_* values were determined: 12.1 ± 5.6, 20.2 ± 12.7, 2.4 ± 1.0, and 5.1 ± 1.6 μM, respectively. The corresponding inactivation rate constants were: 0.10 ± 0.02 min^−1^, 0.11 ± 0.04 min^−1^, 0.07 ± 0.01 min^−1^, and 0.04 ± 0.01 min^−1^, respectively. The inactivation efficiency was determined for each flavonoid as the ratio of the inactivation rate constant and the inhibition constant. Out of all the tested flavonoids, chrysin had the highest inhibition efficiency, which was 0.029 min^−1^ μM^−1^ (Table 1).

### 2.2. Hemochrome-Pyridine Assay

**Figure 3:**
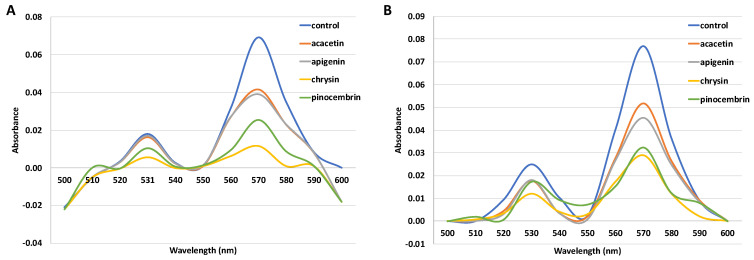
Spectra showing a decrease in heme absorbance in incubations with flavonoids (25 µM) without the addition of SOD and CAT (**A**) and with the addition of SOD and CAT (**B**). Heme concentration determined in the control sample was 0.53 µM (**A**) and 0.60 µM (**B**).

The hemochrome pyridine assay is used to determine the covalent binding of reactive intermediates to the protoporphyrin portion of heme. Under reduced, basic conditions, ferrous forms a complex with pyridine. The absorption maxima of the complex were observed at 531 nm and 570 nm. Incubations containing flavonoids as inhibitors (acacetin, apigenin, chrysin, and pinocembrin) showed a decrease in heme concentration (Figure 3). The assays were then confirmed by additional incubation with catalase (CAT) and superoxide dismutase (SOD) to prevent the possible destruction of heme by reactive oxygen species formed in nonproductive cytochrome P450 cycles. A reduction of heme concentration was also confirmed (Figure 3). Incubation with acacetin reduced heme by 51.12%, apigenin by 54.95%, chrysin by 94.5%, and pinocembrin by 74.73%.

All flavonoids reduced the heme concentration in the assay with and without the addition of SOD and CAT. The residual heme concentration after incubation with acacetin was 48.88%, and, on retesting with the addition of SOD and CAT, was 63.33%. The residual heme concentration after incubation with apigenin was 45.05%, and, on repeated testing with the addition of SOD and CAT, was 55.11%. The residual heme concentration after incubation with chrysin was 2.9%, while after incubation with the addition of SOD and CAT was 5.5%. The second largest reduction of heme concentration was observed in incubations with pinocembrin, which were at 25.3% and 35.5%, without and with the addition of SOD and CAT, respectively (Table 2).

These results indicate that the inactivation of the cytochrome P450 3A4 enzyme is achieved through the adduct formation of reactive flavonoid intermediate with the heme portion of the enzyme. As the results were confirmed with the use of CAT and SOD, it can be concluded that heme destruction was not caused by reactive oxygen species generated in non-productive cycles of cytochrome P450.

### 2.3. Pseudo-Irreversible Inhibition Assay

**Figure 4:**
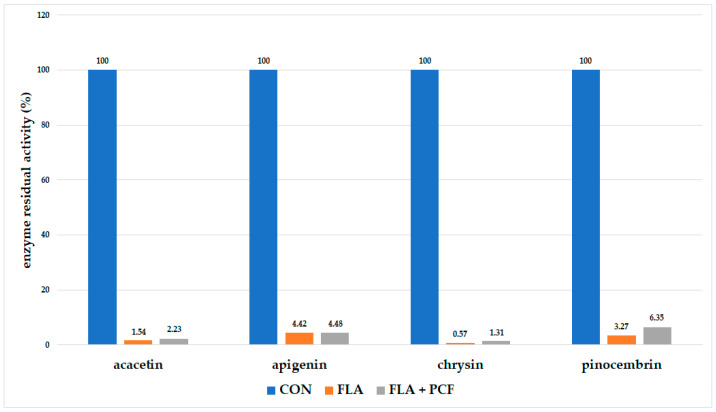
Residual activity of the CYP3A4 enzyme after incubation and dialysis with flavonoid or after incubation and dialysis with flavonoid treated with potassium hexacyanoferrate (CON—control, FLA—flavonoid only, FLA + PCF—flavonoid, and potassium hexacyanoferrate).

In the pseudo-irreversible inhibition assay, samples were incubated with flavonoids and treated with oxidant, after which they were subjected to dialysis. In the case of pseudo-irreversible inhibition, the enzyme activity should be recuperated. In the incubations with all of the tested flavonoids, there was a significant inhibition of enzyme activity after dialysis with and without treatment with an oxidant (Figure 4). In all of the cases, the difference in residual enzyme activity between the flavonoid sample and the potassium hexacyanoferrate sample was statistically insignificant (*p* ≤ 0.05).

As in the previous experiments, the highest reduction of enzyme activity was observed when chrysin was used as an inhibitor. The residual activity of the enzyme after incubation and dialysis with chrysin was 0.57%, and after incubation and dialysis with prior treatment with potassium hexacyanoferrate, it was 1.31% (Figure 4).

In case of direct (reversible) inhibition, this type of experiment would show enzyme activity recovery after dialysis. In case of pseudo-irreversible inhibition, enzyme activity would be recovered after treatment with an oxidant and dialysis. As these were not the case with any of the analyzed flavonoids, it can be concluded that all flavonoids show irreversible inhibition of cytochrome P450 3A4.

## 3. Discussion

It has been shown that some flavonoids can inhibit the activity of the cytochrome P450 enzymes. Šarić-Mustapić et al. [28] and Kondža et al. [29] showed that acacetin, apigenin, chrysin, and pinocembrin inhibit the CYP3A4 enzyme in the assay with testosterone as a marker substrate. As the CYP3A4 enzyme possesses a large active site that can accommodate substrates differently, it is advised to conduct activity assays with another marker substrate such as nifedipine and midazolam [30]. Consequently, we used nifedipine oxidation as the marker reaction of CYP3A4 activity and determined the inactivation kinetic parameters. The values of inhibition efficacy obtained using nifedipine as a marker substrate are of the same order of magnitude when compared to the testosterone assay for apigenin, acacetin, and pinocembrin. Only chrysin showed a fourfold higher value in testosterone [29] when compared with nifedipine assay, i.e., 0.108 min^−1^ μM^−1^ vs. 0.029 min^−1^ μM^−1^. The observed similarities in the inactivation kinetics are in accordance with literature data confirming that nifedipine and testosterone have different binding sites that overlap [31]. Cytochrome P450 3A4 has one of the largest active sites and can accommodate the substrate and the inhibitor at the same time, with one influencing the binding of the other, which could explain the observed differences between nifedipine and testosterone assays.

The clinical implications of reversible inhibitors (i.e., food–drug interactions) can be avoided if the inhibitor is discontinued from the treatment. More important interactions are those that are irreversible, whereas simply discontinuing the use of the inhibitor will not resolve the interaction issues. In this study, chrysin showed the highest inhibition potential, with the lowest *IC*_50_ and *K_i_* values. The molecular docking study of flavonoids binding to cytochrome P450 3A4 has shown a higher binding affinity of chrysin by exposing the B ring to the iron in the active center of the enzyme [28]. Structure-inhibition relationship study has shown that hydroxyl groups at the A ring contribute to the inhibitory effect probably due to ion–ion interactions, while B ring can be non-substituted (pinocembrin, chrysin) or monosubstituted (acacetin, apigenin) for the inhibitory effect to be observed (Figure 1) [28]. Non-substituted flavonoids (pinocembrin, chrysin) are more susceptible to the epoxidation and generation of reactive intermediates that inactivate CYP3A4. The stronger inhibitory effect observed with chrysin is probably due to the rigidity of the structure and the presence of the C2=C3 double bond when compared with pinocembrin.

The importance of these described enzyme kinetics can be observed in the context of food–drug interactions, where the administration of chrysin (20–100 µM) significantly increased the AUC and peak serum concentration (C_max_) of nitrofurantoin in rats [32]. For instance, the relatively weak mechanism-based inhibitor of the CYP3A4/5 enzyme, erythromycin [33], has been reported to cause a moderate interaction with cerivastatin in healthy volunteers (21% increase in AUC of cerivastatin) [34]. Chrysin also has a larger inhibition potential than mibefradil, an antihypertensive drug that was withdrawn due to significant interactions with other medicines. The results of this study suggest that chrysin can be a potent inhibitor and possibly interact with other used medications, changing the AUC and C_max_ of these medications. Chrysin is abundant in propolis (28 g/L), and it is the third main flavonoid in honey 5.3 mg/kg [35]; therefore, a diet rich in these foods has a potential to interact with medicines that are CYP3A4 substrates.

Pinocembrin, a proven irreversible inhibitor of the CYP3A4 enzyme, showed the lowest *k_inact_* value of 0.04 ± 0.01 min^−1^. Its inactivation efficacy was similar to that of acacetin and apigenin (0.01 min^−1^ µM^−1^). Pinocembrin is mostly found in foods such as honey and beverages (tea and red wine), presenting a rich source of this flavonoid [29]. According to these kinetic parameters, caution should be exercised in consuming these pinocembrin sources along with medicines that act as CYP3A4 substrates.

Acacetin showed three times higher *IC*_50_ value than chrysin and five times higher *K_i_* value, while maintaining similar *k_inact_* values as other flavonoids, indicating a lower inhibitory efficacy. Acacetin can mainly be found in plant species such as *Turnera diffusa*, *Robinia pseudoacacia*, and *Betula pendula* [36], but certain foods also present a source of this flavonoid. For instance, kumquat juice is a rich source of acacetin, and acacetin can be found in concentrations of 0.1 mg/100 g of fresh juice [37]. The parent compound of acacetin, apigenin showed the highest *IC*_50_ value and the highest *K_i_* value among the four tested flavonoids. The inhibitory potential of apigenin was confirmed in vivo when the combination treatment of apigenin and paclitaxel led to the increase in oral bioavailability of paclitaxel, which was mainly attributed to enhanced absorption in the gastrointestinal tract through the inhibition of P-glycoprotein and reduced first-pass metabolism of paclitaxel through the inhibition of the CYP3A subfamily in the small intestine and/or in the liver by apigenin [38]. Apigenin is a dominant flavonoid in celery, parsley, and chamomile. Dried parsley has been reported to have the maximum quantity of apigenin, at 45035 μg/g. Additional sources of apigenin are found in herbs such as the dried flower of chamomile, which contain 3000 to 5000 μg/g, and celery seeds, which contain 786.5 μg/g [39].

**Figure 5:**
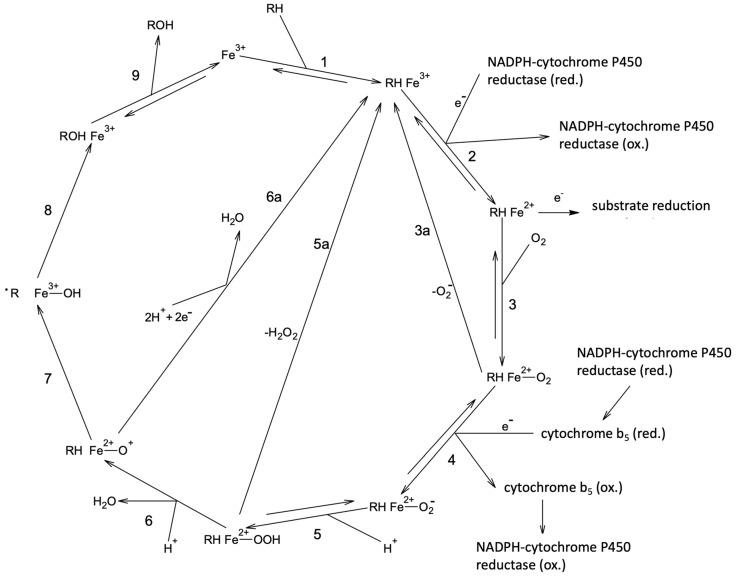
Cytochrome P450 catalytic cycle consists of nine steps. The first step represents binding of the substrate and point of interaction with reversible inhibitors. In the second step, heme iron is reduced to a ferrous form to which pseudo-irreversible inhibitors bind. If an irreversible inhibition is observed, it is usually related to the radical form of substrate (formed in step 7) and/or product (formed in step 8). Reactive oxygen species formed in futile cytochrome P450 cycle (steps 3a and 5a) can cause an enzyme destruction, which can be prevented by the addition of SOD and CAT. The description of steps not related to inhibition is omitted. Prepared as per reference [26].

The reversible inhibition of cytochromes P450 3A4 is related to the first step of the cytochrome P450 catalytic cycle (Figure 5). Most often, the competition between the substrate and the inhibitor in binding to the active site (ferric iron) is observed. In that case, it is advisable to conduct binding experiments (enzyme titrations) and to test it with more than one substrate, as CYP3A4 has a large active site that can accommodate more than one molecule of substrate/inhibitor [26,30]. In this study, we have used nifedipine as a substrate, and the results of inhibition efficiency were of a comparable magnitude with the previously reported assays conducted with testosterone as marker substrate (Table 1).

However, as we have previously shown [28,29] and confirmed here in our conducted experiments, all studied flavonoids show an irreversible inhibition. Irrespective of the type of the irreversible inhibition (pseudo-irreversible or covalent binding of intermediate to heme or apoprotein), inhibition will be most prominent if preincubation, before the addition of a marker substrate, is conducted [26]. In a preincubation, a catalytic cycle is activated by the addition of the generating system for the production of the coenzyme (NADPH), and sufficient time is permitted to observe enzyme inactivation. Thus, all of the irreversible types of inhibition require the reduction of the enzyme to the ferrous form (Figure 5).

As cytochromes P450 are hemoproteins, we have studied the binding of flavonoids to the ferrous form of the active site and the binding of a possible reactive intermediate to the heme [26]. To assess the possible heme adduct formation, a hemochromopyridine assay was conducted. All the tested flavonoids significantly reduced heme concentration in both assays, by more than 50%. Chrysin was, again, the most potent inhibitor, matching the inhibition kinetic values observed in this study. Heme concentration with pinocembrin was five times higher, and, with apigenin, it was almost ten times higher than the one with chrysin (25.3 ± 0.4 and 45.1 ± 1.7%, respectively). This reduction in heme concentrations indicates that the irreversible inhibition of CYP3A4 by the studied flavonoids can be attributed to the covalent binding of a reactive intermediate to the heme. It should be noted that hemes can be destroyed by reactive oxygen species, lowering the results of hemochromopyridine assay. To eliminate this possibility, incubations were conducted in the presence of SOD and CAT (Table 2), confirming the aforementioned conclusion.

The first step in the catalytic cycle of the cytochrome P450 enzyme is the binding of a substrate to a ferric ion. If the inhibitor shows competitive inhibition to the substate, the enzyme activity can be retrieved by dialysis, as the elimination of the inhibitor enables the complete recovery of the enzyme activity [26]. However, if the inhibitor binds to the ferrous iron, the use of an oxidant, such as PCF, preceding dialysis is needed in order to retrieve enzyme activity. If enzyme activity is recovered, inhibition is characterized as pseudo-irreversible [26]. As the enzyme activity could not be recovered after dialysis with or without PCF, none of the tested flavonoids (at 25 μM concentration) act as reversible or pseudo-irreversible inhibitors of the CYP3A4 enzyme. This also confirms that an irreversible inhibition by a covalent binding to either heme or apoprotein is the probable cause of enzyme inactivation.

It should be noted that chrysin caused nearly a complete reduction of heme in the incubation: 97.1% without and 94.5% with SOD and CAT (Table 2); while the results of residual enzyme activity under similar conditions showed the reduction of enzyme activity by approximately 99% (Figure 4). The hemochromopyridine assay registers hemes from all sources in the incubation, i.e., cytochrome P450, as well as cytochrome b_5_. The results for chrysin indicate that a reactive intermediate generated by cytochrome P450 reacted with heme from cytochrome P450 as well as cytochrome b_5_.

Further LC-MS analysis was conducted to determine possible reactive flavonoid intermediates either in a form of heme adduct or trapped using glutathione as a radical scavenger (data not shown). However, we were not able to isolate any of the predicted adducts probably due to the instability of the reactive flavonoid intermediate and/or heme/glutathione adduct. A similar behavior was observed with mibefradil, an antihypertensive, withdrawn from the marker due to drug/drug interactions. Although mibefradil caused the reduction of heme, a reactive intermediate was not established [40].

## 4. Materials and Methods

### 4.1. Materials

Four flavonoids (acacetin, apigenin, chrysin, and pinocembrin) were used in this study (Sigma-Aldrich, St. Louis, MO, USA). Recombinant cytochromes P450 3A4 coexpressed with nicotinamide-adenine-dinucleotide phosphate (NADPH) reductase and cytochrome b_5_ in baculosomes were purchased from Thermo Fisher Scientific (Waltham, MA, USA). Based on cytochrome P450 carbon monoxide assay [41], the content of CYP3A4 enzyme declared by the manufacturer was confirmed to be 1 μM. Glucose-6-phosphate (G6P), glucose-6-phosphate dehydrogenase (G6PDH), and β-nincotinamide-dinucleotide phosphate disodium salt (NADP^+^) were purchased from Sigma-Aldrich. Potassium phosphate (p.a.) and dichloromethane (p.a.) were purchased from Kemika d.d. (Zagreb, Croatia), formic acid (85%, p.a.) from Semikem d.o.o. (Sarajevo, Bosnia and Herzegovina), and methanol used for reagent dissolution and chromatography from Merck KGaA (Darmstadt, Germany). Ultrapure water was used in the incubation mixtures and chromatography. Potassium dihydrogen phosphate (Kemika d.d.) was used to prepare potassium phosphate buffer pH 7.85; pH was adjusted using sodium hydroxide purchased from Semikem d.o.o. Nifedipine (Sigma-Aldrich) and oxidized nifedipine (European Directorate for Quality of Medicines, Pharmacopoeia, 10th edition, Strasbourg, France) were used in the study of residual enzyme activity, as well as in inactivation kinetics. Testosterone (Sigma-Aldrich) and 6β-hydroxytestosterone (Cayman Europe, Tallinn, Estonia) were used for assays in pseudo-irreversible inhibition. Troleandomycin and diltiazem were obtained from the Agency for Medicinal Products and Medical Devices (Zagreb, Croatia). Pyridine (p.a.) (Semikem d.o.o.), bovine hemin (Sigma-Aldrich), and dimethylsulfoxide (Semikem d.o.o.) were used in the hemochromopyridine assay. Potassium hexacyanoferrate (Siegfried AG, Zofingen, Switzerland) was used in the study of reversible and pseudo-reversible inhibition. Superoxide dismutase (SOD) (Sigma-Aldrich), catalase (CAT) (Sigma-Aldrich), and hydrochloric acid (36%, p.a.) (Semikem d.o.o.) were used in the binding specificity assay. Enzyme incubations were performed in a water bath (Inkolab, Zagreb, Croatia). Assay of residual enzyme activity and flavonoid inactivation kinetics were performed using high performance liquid chromatography coupled with UV-Vis detection (HPLC UV-Vis, Agilent 1100, Agilent Technologies, Santa Clara, CA, USA). Residual activity calculations and inhibition kinetics parameters were made using the program R (The R Project for Statistical Computing, Vienna, Austria) and Microsoft Excel (Microsoft, Redmond, WA, USA).

### 4.2. Methods

#### 4.2.1. Determination of Enzyme Kinetics and Residual Activity

Enzyme incubations were performed in triplicate, with mechanical stirring in a water bath at 37 °C. Aliquots of flavonoids with a final concentration of 1 μM were dissolved in methanol, transferred to glass tubes, and evaporated to dryness, except in control samples without the inhibitor (flavonoid). After the evaporation of the solvent, an incubation mixture was prepared to a volume of 100 μL, consisting of 5 pmol CYP3A4 enzyme, 50 mM potassium phosphate buffer (pH 7.4), and ultrapure water. An NADPH generating system composed of 0.1 M G6P: 10 mg/mL NADP^+^: 1000 IU/mL G6PDH = 50:25:1 (*v*/*v*/*v*) was prepared immediately before use. This generating system contains glucose-6-phosphate dehydrogenase that regenerates NADP^+^ into NADPH, keeping the concentration of the cytochrome P450 coenzyme (NADPH) constant in the incubation. The addition of the generating system (15% of the volume in the final incubation, *v*/*v*) started the reaction. Nifedipine was used to test out the residual enzyme activity at a final concentration of 200 μM. The reaction was stopped using 1 mL of ice-cold 1% solution of formic acid in dichloromethane. The samples were mixed, then centrifuged for 10 min on 1900g (Rotofix 32, Westfalia, Germany). After centrifugation, two layers were formed (water and organic layer). 850 µL of the organic layer was transferred to cuvettes and evaporated. The analyte was then dissolved in 30 µL of methanol. An Agilent Zorbax SB C18 column (4.6 × 250 mm, 3 μm, Agilent Technologies, Santa Clara, CA, USA) was used to analyze the samples by HPLC. The mobile phase was composed of methanol and water in a ratio of 64:36. The analysis was isocratic. The flow was set at 1.0 mL min^−1^. The injection volume was set at 10 µL. Nifedipine was used as a marker substrate. The reaction observed was the oxidation of nifedipine. Chromatograms were recorded at 254 nm. The duration of the analysis was set at 25 min. The retention time of nifedipine is 7.1 min and that of oxidized nifedipine is 10.8 min [42]. In both cases, the amount of product obtained was observed as the amount of area under the curve (AUC) relative to the control sample. For all the flavonoids, half maximal inhibitory concentration (*IC*_50_), constant of inhibition (*K_i_*), constant of inactivation speed (*k_inact_*), and inactivation efficacy (*k_inact_*/*K_i_*) were determined. Troleandomycin was used as a positive control—irreversible inhibitor of cytochrome P450 3A4. 25 μM concentration of troleandomycin reduced enzyme activity to 35 ± 5%.

#### 4.2.2. Hemochromopyridine Assay

The hemochromopyridine assay was used to assess possible heme destruction by reactive intermediate form in cytochrome P450 cycle and was performed according to the method described by Flink and Watson [43] and Paul et al. [44], with some modifications. A calibration curve was established using hemin dissolved in dimethylsulfoxide (0.6 to 0.1 μM). Spectra were recorded at a wavelength of 500 to 600 nm. Incubation mixtures with 25 μM inhibitor (flavonoid) were prepared to a volume of 200 μL. The reaction was started by the addition of an NADPH generating system (for composition, see Section 4.2.1), and the incubations lasted for 30 min. The reaction was stopped after half an hour by the addition of pyridine (final concentration 0.83 M) and sodium hydroxide (final concentration 0.06 M). The samples were recorded on a spectrophotometer (UV-1280, Shimadzu Corporation, Kyoto, Japan) within 1 min of the addition of an alkaline solution, due to the instability of pyridine hemochromogen under basic conditions [44]. Heme concentration in the samples was calculate based on the prepared calibration curve. This assay was performed in triplicate. The incubations were repeated using catalase and superoxide dismutase (5 IU each) in the incubation mixture to prevent the generation of hydrogen peroxide through futile catalytic cycle.

#### 4.2.3. Pseudo-Irreversible Inhibition Assay

The aim of this assay is to test out the formation of covalent complexes with ferrous iron (Fe^2+^), by assessing a recovery of enzyme activity after dialysis with or without the addition of an oxidant. Three types of experiments were performed for each flavonoid: incubation mixture without flavonoids (control), incubation mixture with flavonoid, and incubation mixture with flavonoid to which oxidant was added after incubation. Each incubation with the inhibitor was performed for 30 min using the same experimental settings as described above (Section 4.2.1). After incubation, the samples were transferred to dialysis cartridges. Aa oxidant—20 mM solution of potassium hexacyanoferrate was added to one of the incubations before dialysis [45]. The cassettes (Slide-A-Lyzer Dialysis Cassettes, Thermo Fisher Scientific, Waltham, MA, USA) were immersed in 50 mM potassium phosphate buffer solution (pH 7.4) for 30 min (dialysis solution was exchanged three times). After dialysis, the samples were transferred from the cassette back to the glass tube, and the residual enzyme activity was assessed using testosterone as a marker substrate (200 μM final concentration). As the NADPH generating system was also dialyzed, it was again added to the incubations to initiate enzyme reaction. The samples were incubated for 30 min with the same settings (described under Section 4.2.1). The reaction was terminated by the addition of an ice-cold solution of formic acid in dichloromethane. The samples were analyzed by HPLC. Diltiazem, a known pseudo-irreversible inhibitor of cytochrome P450 3A4, was used as a positive control to evaluate oxidation to a ferric form (a complete enzyme activity recovery was observed).

#### 4.2.4. Results Processing

A one-sided *t*-test would be used to assess the statistical significance in the differences between samples and controls, based on measurements of residual activity. A nonlinear equation was used to calculate the *IC*_50_ value. The Michaelis-Menten equation was used to determine the inactivation constant and the inactivation rate of the inhibitor. Statistical processing and graph preparations were done using Program R (The R Project for Statistical Computing, Vienna, Austria) and Microsoft Excel (Microsoft, Redmond, WA, USA).

## 5. Conclusions

Acacetin, apigenin, chrysin, and pinocembrin inhibit the CYP3A4 enzyme activity in vitro. Chrysin is the most potent enzyme inhibitor, with the lowest *IC*_50_, *K_i_*, *k_inact_* values and the highest inactivation efficacy. All flavonoids reduced the heme concentration of the enzyme, confirming that this is an irreversible inhibition by reactive intermediate that cannot be prevented by the addition of SOD and CAT. None of the tested flavonoids act as reversible or pseudo-irreversible inhibitors of the CYP3A4 enzyme at 25 μM concentration, as the enzyme activity could not be recovered with dialysis with and without the addition of potassium hexacyanoferrate. As these flavonoids can abundantly be found in various foods such as fruits, vegetables, spices, teas, and red wine, there is a possibility that they can interfere with various xenobiotics that are CYP3A4 substrates. Further in vivo studies are needed to completely investigate the possibilities of such food–drug interactions, as well as the possible contribution of other enzymes and transporters in the interactions.

## Figures and Tables

**Table 1:** Basic kinetic parameters of CYP3A4 inhibition by individual flavonoids (nifedipine (NIF), used as marker substrate, and the results expressed as mean values of triplicates). The results are compared with the assays in which testosterone (TSN) was used as marker substrate (taken from reference [29]).

	Acacetin		Apigenin		Chrysin		Pinocembrin	
Parameter	NIF	TSN	NIF	TSN	NIF	TSN	NIF	TSN
*IC*_50_ (µM)	7.5 ± 2.7	10.9 ± 0.3	8.4 ± 1.1	11.4 ± 0.4	2.5 ± 0.6	0.6 ± 0.5	4.3 ± 1.1	5.0 ± 0.6
*K_i_* (µM)	12.1 ± 5.6	6 ± 3	20.2 ± 12.7	1.5 ± 0.8	2.4 ± 1.0	0.6 ± 0.3	5.1 ± 1.6	1.2 ± 0.3
*k_inact_* (min^−1^)	0.10 ± 0.02	0.036 ± 0.006	0.11 ± 0.04	0.11 ± 0.01	0.07 ± 0.01	0.065 ± 0.005	0.04 ± 0.01	0.018 ± 0.001
*k_inact_*/*K_i_* (min^−1^ µM^−1^)	0.008	0.006	0.005	0.073	0.029	0.108	0.008	0.015

**Table 2:** Heme concentration after flavonoid incubations expressed as percentage to the control incubation without an inhibitor.

Flavonoid	Heme Concentration (%)	Heme Concentration with the Addition of SOD and CAT
acacetin	48.8 ± 0.4	63.3 ± 0.5
apigenin	45.1 ± 1.7	55.1 ± 2.9
chrysin	2.9 ± 0.1	5.5 ± 0.5
pinocembrin	25.3 ± 0.4	35.3 ± 1.2

## Data Availability

The data that support the findings of this study are available from the first author, M.K., or corresponding author, M.B., upon request.
